# Static and Dynamic Evaluation of a Winding Deformation FBG Sensor for Power Transformer Applications

**DOI:** 10.3390/s19224877

**Published:** 2019-11-08

**Authors:** Aguinaldo Goes de Melo, Daniel Benetti, Luiz Alkimin de Lacerda, Rodrigo Peres, Claudio Floridia, Artur de Araujo Silva, Joao Batista Rosolem

**Affiliations:** 1 COPEL Geração e Transmissão, Curitiba 81200-240, PR, Brazil; aguinaldo.melo@copel.com (A.G.d.M.); daniel.benetti@copel.com (D.B.); 2 LACTEC - Instituto de Tecnologia para o Desenvolvimento, Curitiba 81530-180, PR, Brazil; alkimin@lactec.org.br; 3 CPQD Research and Development Center in Telecommunications, Campinas 13086-902, SP, Brazil; rperes@cpqd.com.br (R.P.); floridia@cpqd.com.br (C.F.); arturs@cpqd.com.br (A.d.A.S.)

**Keywords:** power transformer, short circuit, winding, deformation, pressure sensor, FBG

## Abstract

Power transformer is the most important and expensive equipment used in the electric power industry. Fiber Bragg grating (FBG) sensors has stood out as a flexible and particularly suitable tool for power transformer monitoring being a passive and dielectric sensor element. In this work we evaluated the performance of FBG pressure sensors developed to monitor the static and dynamic pressure in high voltage winding transformers during events such as short-circuit and inrush current. Two types of sensors packaging materials were evaluated in laboratory: polyether ether ketone (PEEK) and transformerboard (TB). The sensors have been tested for high intensity and short duration impacts similar to those occurring in short circuits. In addition, we evaluated the time response of sensors using an interrogation system with a 5 kHz sweep in order to analyze the short circuit response time properly. The results pointed that FBG pressure sensors using PEEK and TB are suitable for transformer winding monitoring. The static sensitivity obtained to PEEK based sensors was 0.911 pm/N, in the range of 800 N to 1500 N. This sensitivity is 4.47 higher than TB based sensors sensitivity. Dynamical tests performance showed an excellent repeatability for both sensors, in agreement with static observation.

## 1. Introduction

The power transformer represents the most strategic and expensive equipment of the power transmission system. Failure in a transformer can lead to power outages, with inconvenience and damage to consumers. In this scenario, it is essential to monitor operational parameters during lifespan of the power transformers, especially the ones used in high voltage transmission lines, given their impact in the stability and reliability of the entire electric system [1].

The main cause of mechanical damage to an operating transformer is the high electromagnetic force (EMF) that originates from intense currents flowing in the transformer windings [2]. Such currents may be induced by internal faults in the transformers or by external faults in the network to which the transformer is connected. Such situations result in huge EMFs in short periods of time that are sufficient to mechanically deform or damage the windings. There are some typical types of mechanical damages that may appear in transformer windings due to high currents of short circuit faults, such as axial displacement, bending or tilting of windings due to axial forces and winding deformation due to radial forces.

Thus, it is important to know the value of the maximum force exerted on the windings in short circuit situations for the correct design of a mechanical strain sensor. In the literature there are many works that calculated, simulated or eventually measured this force [3,4]. According to the studies of Zhang et al. [2] the dynamic forces on the windings of a power transformer can reach peaks of 100 kN or a pressure of 14 MPa when a short circuit occurs.

For the detection of deformations caused by mechanical stresses in a transformer there are indirect tests that can detect changes in the coil geometry, but until now there are no means to measure the stresses imposed on the transformers and possible windings displacements when they are subjected to fault currents in operational conditions.

With the advent of sensor technology using optical principles, it has been possible to directly measure the displacements in power transformer windings. The main advantage of this technology is that optical fibers are passive and present immunity against electromagnetic interference. These two characteristics are very important in the internal transformer environment. Direct measurement of mechanical strains in the transformer windings using a single fiber optic sensor technology can be a powerful tool for early detection of internal problems such as the occurrence of hotspots and the displacement or loosening of winding insulating parts due to stresses arising from short circuit currents. These measurements can also record the intensity and history of these transient events.

Fiber Bragg grating (FBG) sensors has stood out as a flexible and particularly suitable tool for power transformer monitoring. Many studies were published describing FBG as a partial discharge [5,6,7,8,9,10,11], gas [12,13] and for a temperature sensor [14,15,16,17] in power transformers. However, there are few studies for deformation or pressure FBG sensors. 

Liu et al. [18] described a study to identify the dynamic strains in a transformer iron core using FBG sensor, while Kuhn et al. [19] described an FBG axial pressure sensor used for monitoring transformer windings.

In this work the performance of FBG pressure sensors developed for monitoring static and dynamic pressures in high voltage winding transformers due to short-circuit and inrush currents is evaluated. Two types of sensor packaging materials were evaluated in laboratory: transformerboard (TB) and polyether ether ketone (PEEK). These materials are suitable for power transformer sensing devices due excellent mechanical, electric, thermal and chemical characteristics. The proposed new geometrical design of the deformation sensor has an inverted dome when compared with former reference [18]. A flat cylinder on the top of the sensor is also introduced. Both approaches increase the sensitivity of the sensor and inverted dome is also safer for the optical fiber as no force acts in it laterally. In addition a new technique that permits to simulate the effect of transient currents on the sensor has been introduced to perform the impact test. Finally, TB was used and tested for the first time to our knowledge as deformation sensor base. TB is a material already employed in the transformers, but was used here as part of the sensing element, a sensed shim.

The sensors were stressed in typical short circuit winding pressures. In addition, the time response of sensors was evaluated using a 5 kHz interrogation system in order to be able to analyze the short circuit response time properly. The results pointed that FBG pressure sensors using PEEK and TB are suitable for transformer winding monitoring. The static sensitivity obtained to PEEK based sensors was 0.911 pm/N, in the range of 800 N to 1500 N. This sensitivity is 4.47 times higher than TB based sensors. Dynamical tests performance showed an excellent repeatability for both sensors, in agreement with static observation.

## 2. Materials and Methods

### 2.1. Methods

A FBG is a point type optical sensor. It is constructed by creating a distributed Bragg reflector or a periodic variation in the fiber core refractive index on a short segment of an optical fiber. This structure reflects specific wavelengths of light while transmitting others. The wavelength reflected in the Bragg reflector (Bragg wavelength) is defined by λ_B_ = 2n_e_.Λ, where n_e_ is the effective refractive index of the grating and Λ is the granting spacing period. When a strain is applied to the FBG, the grating period is changed and consequently the Bragg wavelength also changes linearly. This dependence of the FBG wavelength on mechanical strain has made it very interesting for use in many applications. Many types of substrates and materials are adapted to FBG to build different types of sensors.

The main advantage of a FBG for sensing applications is the ability to directly transform the variation of a physical parameter into a variation of optical quantity, in this case wavelength. When compared to conventional sensors, such as a thermocouple, for example, FBG sensors have many advantages: lightweight, compact, dielectric (nonconductive), passive (do not require external power), wide bandwidth and they have high sensitivity. FBG sensors are also corrosion/oxidation resistant and can receive various types of coatings.

A well-known property of FBG sensors is that they can be connected in series on the same fiber creating a sensor network, as each FBG sensor reflects in a specific wavelength range by construction choice [20]. This feature is very important as it allows the elements to be connected to one single interrogator equipment, thus reducing the total system cost.

**Figure 1:**
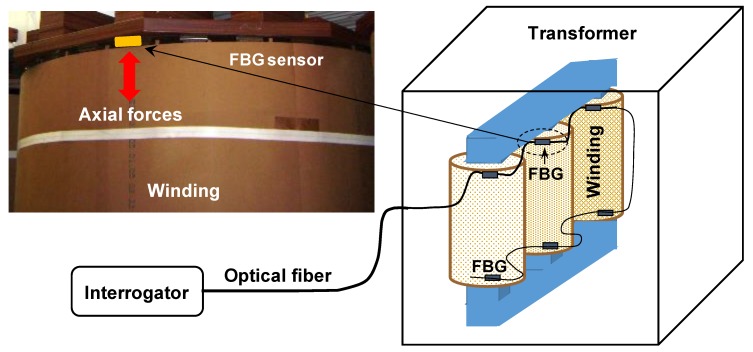
Winding deformation sensing system conception showing how the Fiber Bragg Grating (FBG) sensors are fixed in the in the outer winding and in outer structure, due to ease of installation in these points.

The proposed transformer deformation sensing system is based in a FBG sensors network connected in series inside the power transformer. Basically, the FBG deformation sensors are inserted in the top or in the base of transformer windings, as it is shown in Figure 1. The placement of the sensors in a real transformer is proposed to occur in the outer winding and in outer structure, due to ease of installation in these points. They would be installed in usual points where there exist shims that would be replaced by sensed shims. In a real installation, the load position will define an initial wavelength point for the FBG sensor and it will respond as a variation around this wavelength. In other words, the initial wavelength will be known at the time of installation, as actually occurred in the performed simulated experiments.

In this case the deformation measured by the sensors is due to the axial forces. A single interrogator can analyze the signals of many deformation sensors connected in series, but one of the most important specifications for interrogator in this particular application is its response time.

### 2.2. FBG Deformation Sensor Shape

**Figure 2:**
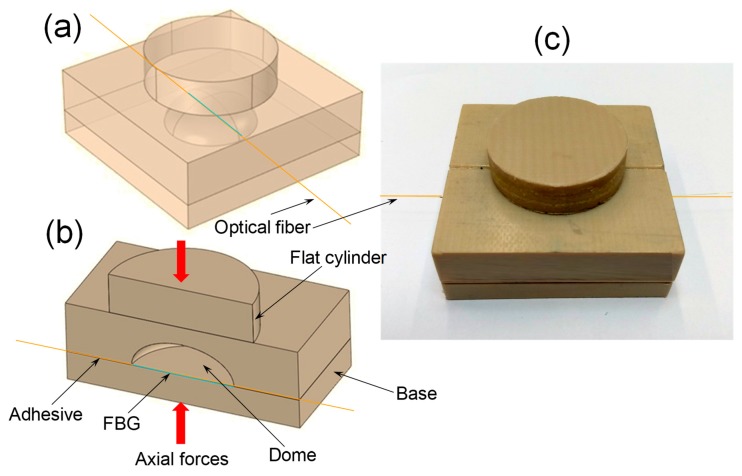
(**a**) 3D shape of FBG winding deformation sensor, (**b**) cross section FBG winding deformation sensor showing the dome and the flat cylinder and (**c**) a real conception of the FBG winding deformation sensor.

The substrate material where the FBG is fixed is very important in the sensor performance. Two important issues regarding this substrate are the shape and the material type. The shape is dependent of where the sensor is placed in the windings. In order to simplify the introduction of the sensor in a future transformer fabrication the sensor shape as shims for the windings were adopted. However, some modifications were inserted in the shim shape in order to obtain a better sensitivity when axial forces are produced in the windings. Figure 2 shows the sensor conception. This shape is an evolution of the shape described in [18]. The sensor consists in a rectangular block of a special material where an empty dome is designed inside the block. In the block top a flat cylinder shape element is fixed, which concentrates the axial forces towards the dome positioned just below. The FBG fiber with polyimide coating was fixed at the bottom of the dome using a high temperature commercial epoxy adhesive, which also contained bisphenol-A-(epichlorohydrin). The cure was carried out in ambient temperature during 24 h. This adhesive resists to water, oils and temperatures up to 120 °C. The adhesive also does not suffer contraction, dilatation or deformation and is suitable for a wide variety of materials, such as: glass, wood, ceramics and rubber, among others. It was tested for PEEK and TB with good results.

Below the rectangular block containing the empty dome there is a board base that protects the FBG. All parts of the substrate were made of the same type of material.

### 2.3. FBG Deformation Sensor Materials

Any material or component introduced into transformers should not cause undesirable effects such as partial discharges, for example, and in addition they have to operate with no degradation for many years in typical temperature of 85 °C, but reaching 120 °C occasionally. The sensors were constructed using two different materials. The first material was the transformerboard (TB), which is a kind of electrical insulation cellulose that has been used in the manufacture of transformer winding shims for many years. The second material was PEEK, a kind of organic thermoplastic polymer. Both materials have optimum electrical insulation characteristics, which should ensure that the dielectric performance of the instrumented transformer would not be affected. The rest of the sensor materials consist of the optical fiber (silica) containing the FBGs and their coating (polyimide). In Table 1 the typical properties of PEEK [21], TB [22] and optical fiber [23,24,25] materials are shown.

In this work the goal of the load cell was to measure the axial component of the force generated by transient electric current of each transformer winding, in addition to its weight and the manufacturing pressure. The sensors were housed in the windings as wooden shims attending the usual dimensions with the load cells embedded in the shims, resulting in an instrumented shim.

Due to FBG’s sensitivity to elongation, the optical sensor design was based on the bending load cell as shown in Figure 2a. A drawing of the FBG sensor package based on this principle is presented in Figure 2b. As can be seen from Figure 2b, the operation of the sensor was based on the thin region deformation of the FBG support material (concavity), where the FBG must be installed to capture such an effect. This structure can be satisfactorily modeled by a two-beam, as the stiffness of the other regions of the block is much greater than that of the region of interest. The sizing of the sensor follows the prismatic beam design methodology according to the classical theory of materials resistance. The design parameters were: length width (b); height (h), force distributed in the contact region (p), modulus of elasticity (E) and the yield strength (σ) of the material.

## 3. Results

**Figure 3:**
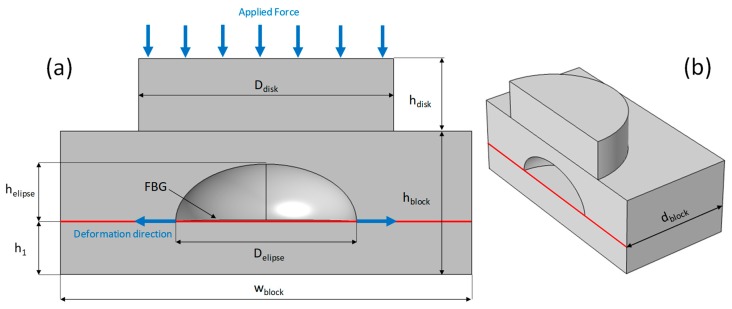
A 3D model of the strain sensor considered in the experiments with its characteristic dimensions. (**a**) Front view and (**b**) isometric view (half sensor).

The following results refer to sensors manufactured with the two distinct types of materials: PEEK and TB. A 3D model of the sensors considered in this work, as well as their parameters, is presented in Figure 3, where in Figure 3a the sensor is shown in a sectional front view and in Figure 3b seen in an isometric view. Table 2 contains the parameter values shown in Figure 3.

**Figure 4:**
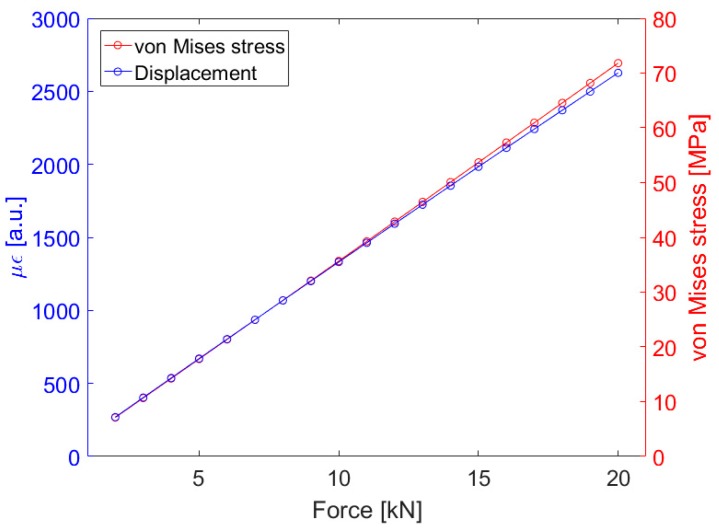
COMSOL simulation results for PEEK sensor. The blue curve (left) shows the strain values (με) and the red curve (right) shows the maximum Von Mises stress values in the sensor structure.

Before performing the experimental measurements, a computational study was conducted using the COMSOL Multiphysics finite elements method (FEM) software. This study aimed to evaluate how the proposed model geometry deforms when a constant force is applied to the upper face of the disk located on the upper part of the sensor. For a PEEK sensor, the simulation result is shown in Figure 4, where the left y-axis (blue) shows the deformation values in με as a function of the applied force. These values of displacement obtained in COMSOL were performed where the FBG was positioned, i.e., the variation of the D_elipse_ parameter was measured in this situation. In Figure 4 the right y-axis (red) shows the Von Mises stress values as a function of the applied force, the entire solid volume was considered and the maximum stress value for each applied force was measured, these forces and deformations refer to arrows indicated in Figure 3. Since the material data sheet [21] indicates that the elastic limit of PEEK is 87 to 95 MPa, the stress values calculated in the simulation did not reach this limit, thus allowing the sensor to deform elastically in its operation.

**Figure 5:**
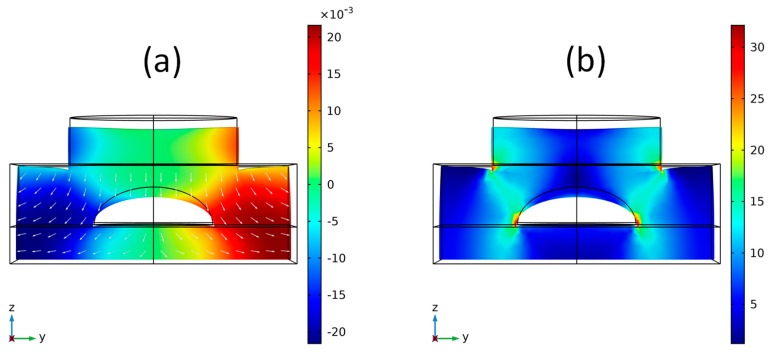
Surface graphics resulting from COMSOL simulation for PEEK sensor. (**a**) Deformation surface graph (mm) and (**b**) Von Mises stress graph (MPa).

Figure 5 presents the contour plots that were obtained in the simulations. The graph in Figure 5a shows the displacement in millimeters suffered by the sensor in the presence of a force of 10.000 N. In addition, the graph of Figure 5b shows a von Mises stress surface graph in MPa that the structure presents when pressed by a force of 10.000 N. The stresses and deformation shown in these figures were related to the quantities of Figure 4. The important parameter was the deformation suffered by the fiber and the upper pressure was a reference for the obtained strain in this FBG position.

These computer simulations indicated that the planned structure would work in elastic regime and the deformation values would be supported by the optical fiber attached to the structure. Next, we performed the static tests.

### 3.1. Static Tests

**Figure 6:**
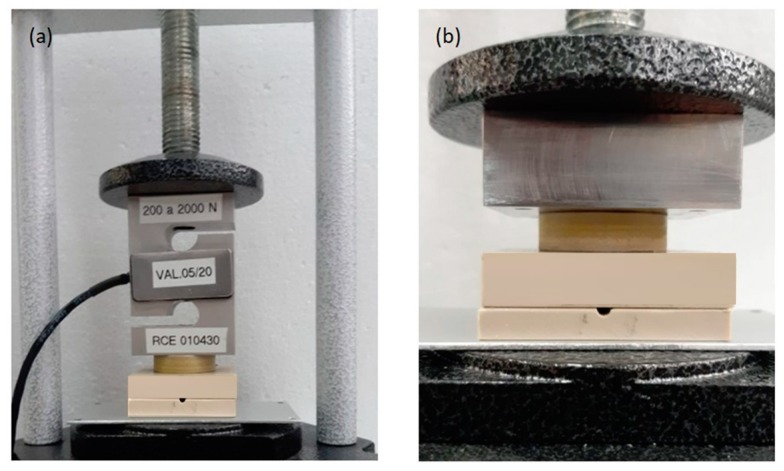
Positioning of the sensor in the hydraulic press machine to perform the static tests. (**a**) Sensor in series with a commercial strain gauge and (**b**) without the presence of the commercial strain gauge. See the online video V1 about this measurement.

To perform the static tests the sensors were positioned in a hydraulic press machine in two situations as shown in Figure 6, one in which the sensor is in series with a commercial S-type strain gauge (Figure 6a) and another in which the sensor was positioned without the strain gauge (Figure 6b). The S-type load cells were used to measure tensile or compressive forces. The spring element was located in the centre beam of the load and translated force into an electrical signal.

These two set-ups were made with the purpose of measuring forces below 1000 N. Since this was the smallest value that could be measured through the hydraulic pressure gauge, it was decided to use the commercial strain gauge with a more sensitive measuring range below 1000 N.

Knowing that the force applied to the sensor was concentrated on the upper disk face, diameter D_disk_, to convert the applied force value to pressure, consider Equation (1):

(1) $$P\left(MPa\right)=\frac{F\;\left(N\right)}{\pi {\left(\frac{D_{disk}\left(mm\right)}{2}\right)}^{2}}.$$

**Figure 7:**
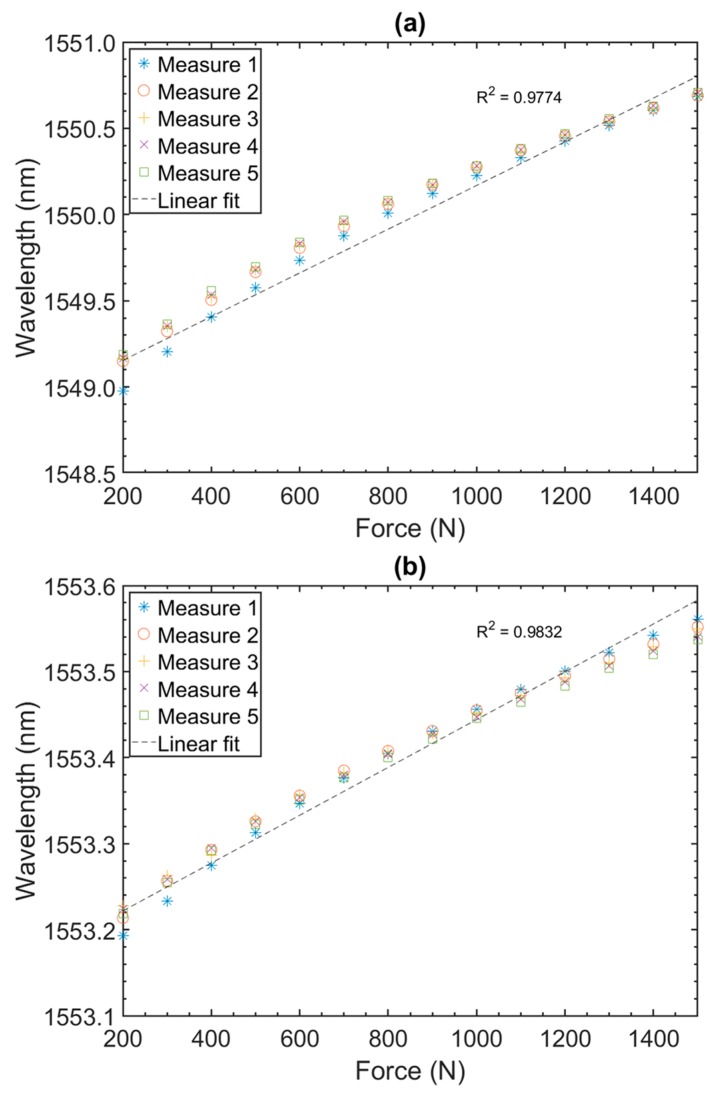
Static test results performed on PEEK (**a**) and TB (**b**) sensors for force values below 2000 N.

Using a commercial interrogator (si155 Hyperion from Micron Optics) with a 5 KHz scan rate connected with the sensor the initial tests were performed with applied forces below 2000 N using a reference S-type strain gauge. The results of the tests are shown in Figure 7. Figure 7a referring to the PEEK sensor and Figure 7b referring to the TB sensor. The difference in wavelength between the maximum and minimum value for the five measurements reported in Figure 7a (PEEK) was 212 pm, and above the 800 N, where behavior was more linear, this variation reduced to 72 pm. Discarding the first measurement due the mechanical accommodation behavior of the system, these differences reduced to 52 pm and 20 pm, respectively. The sensitivity in the linear part of the plot (>800 N) was 0.911 pm/N.

The difference in wavelength between the maximum and minimum value for the five measurements reported in Figure 7b (TB) was 35 pm, and above the 800 N, where behavior was more linear, this variation reduced to 24 pm. The sensitivity in the linear part of the plot (>800 N) was 0.204 pm/N. Thus, results of Figure 7 shows a sensitivity 4.47 times higher for the PEEK material compared to the TB sensor. In addition, good repeatability was noted in both cases, especially if one discards the first measurement made in PEEK, in both cases above 800 N variation was around ~20 pm between maximum and minimum wavelength value.

**Figure 8:**
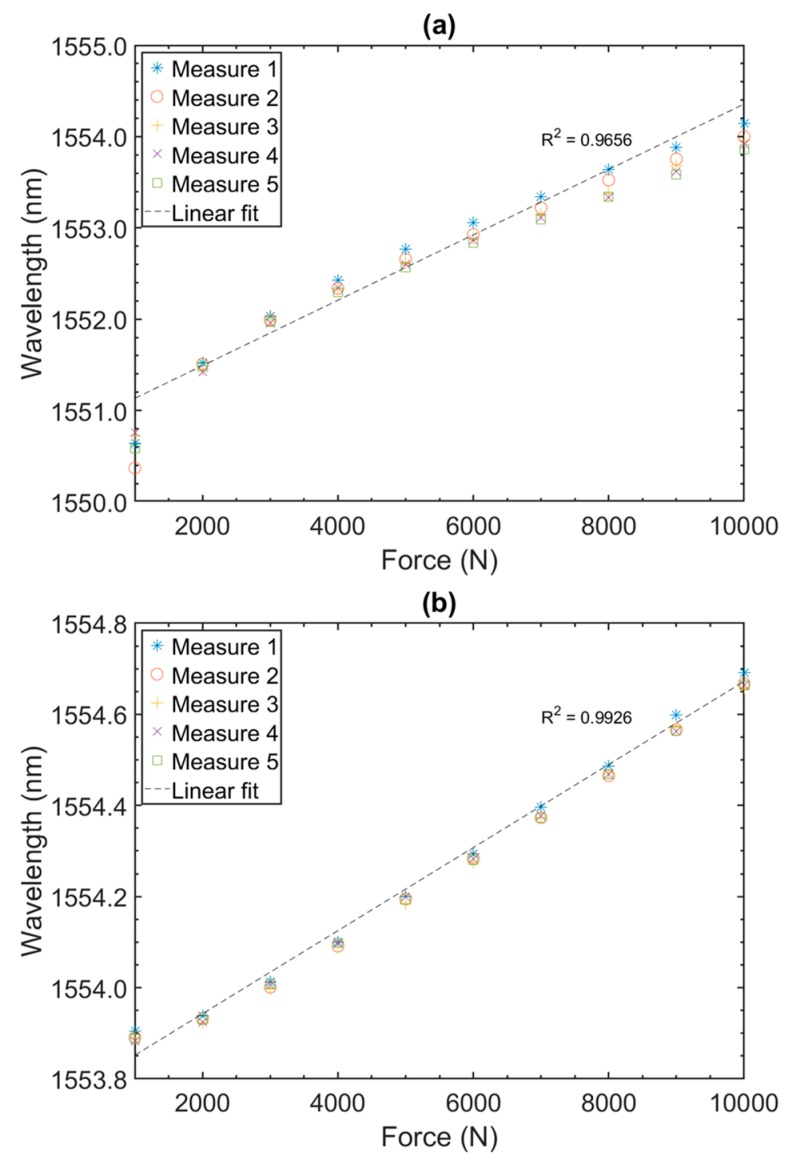
Static test results performed on PEEK (**a**) and TB (**b**) sensors for force values from 1000 to 10,000 N.

Tests were also performed for higher forces from 1000 to 10,000 N (Figure 8). The difference in wavelength between the maximum and minimum value for the five measurements reported in Figure 8a (PEEK) was 390 pm. Above 3000 N, where behavior was more linear, this variation reduced to 305 pm. The sensitivity in the linear part of the plot (>3000 N) was 0.278 pm/N.

The difference in wavelength between the maximum and minimum value for the five measurements reported in Figure 8b (TB) was 36 pm. The sensibility in the linear part of the plot (>2000 N) was 0.0933 pm/N.

Again, the PEEK material sensor (Figure 8a) was more sensitive compared to the TB sensor (Figure 8b). However, in this time, it was 2.98 better. In addition, sensitivity compared with the previous range (800 to 1500 N) was reduced by a factor of 3.28 for the PEEK and by a factor of 2.19 for TB, this fact could be attributed to nonlinear behavior of the transducers for all range of pressure as usual in strain gauge equipment’s [26]. In addition, repeatability of measurements was maintained even at high forces, however TB showed a better performance (36 pm) compared with PEEK (390 pm) for this regime of higher forces.

We would like to note that, as can be seen in Figure 7 and Figure 8, the sensors had a small quadratic deviation from the linear behavior over large ranges, which were believed to come from the mechanical hysteresis of the material. For small ranges linear behavior was predominant. As a comparison, commercial strain gages were used based on a trade-off between range and desired sensitivity. In other words, the developed sensor must have its range set according to the desired application. In a real installation there was an initial pre-stress of the sensors when attached to the coils, and the excursion would be around this point.

### 3.2. Dynamic Impact Tests

**Figure 9:**
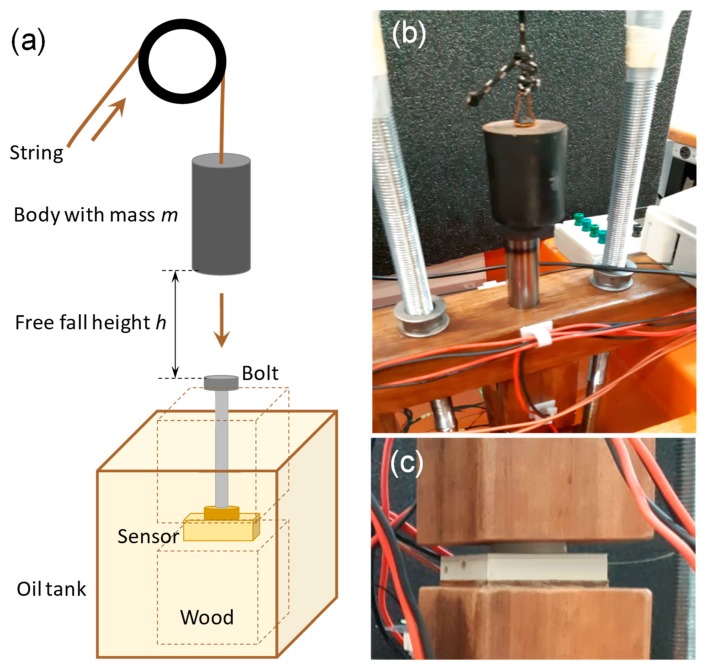
(**a**) Dynamic test set-up arrangement that aims to simulate the shims loading conditions within a power transformer. (**b**) Free-fall body and (**c**) deformation sensor.

Dynamic impact tests were performed in the sensors to evaluate their sensitivity and time response to the impulsive forces that transformer coils exert in the event of an electrical current surge. Since to perform this evaluation in a power transformer in operational regime is very complex a special and innovative test set-up was prepared to simulate the mechanical conditions imposed to the shims within a power transformer. This setup can be seen in Figure 9. Basically, the test consists of the free-falling a body of mass m onto the sensor from a height h. Figure 9a shows the set-up arrangement, Figure 9b shows the body that strikes a bolt which transfers the load to the sensor shown in Figure 9c. An oil tank was used in dynamical tests to proper simulate the same conditions of a real transformer, which was completely embedded in mineral oil to ensure electrical insulation. Thus, sensors where placed in full oil immersion as would be in a real installation.

To achieve force values proportional to those occurring in a real transformer [2], the impulsive force principle was used. Assuming that a body is free-falling one needs to know the impact force of this body on the ground. The velocity v_i_ of this body a moment before impact is known to be given by the Torricelli equation $v_{i}=\sqrt{2\mathrm{gh}}$, where g is the acceleration of gravity and h is the free fall height. To calculate the impact force, we first calculated the deceleration of the body when in contact with the ground as shown in Equation (2).

(2) $$a=\frac{\Delta v}{\Delta t}=\frac{v_{f}-v_{i}}{\Delta t}.$$

Since the final velocity v_f_ is zero and ∆t is the body’s contact time with the ground, the impact force is:

(3) $$F=\mathrm{ma}=m\frac{\sqrt{2\mathrm{gh}}}{\Delta t},$$

where m is the mass of the body. Looking at Equation (3), it can be seen that a dynamic force in the order of tons with a weight of a few kilograms can be achieved by choosing a sufficiently large drop height h.

In this way, a body with a mass m = 1.2 kg was suspended with a string and a pulley so that it could be thrown from a known height against the FBG-based strain sensor. Several dynamic tests were performed for three different sensors. The first sensor (named S05) used TB material, the sensor S08 and S09 used PEEK material.

The pressure calculated from the ball-fall technique was obtained from the upper part of the sensor (disk), as the impact came from this direction in the proposed experiment. In a real experiment there would be two contact surfaces and two pressures involved, we were using as reference the bigger pressure that came from the minor area of the sensor. Thus, the upper pressure was a reference for the obtained strain in the FBG position.

Ten measurements were made by dropping the weight from a known height, from 10 cm to 50 cm. To obtain the sensors data a 5 kHz scan rate interrogator was used. This scan rate was considered high for a large bandwidth FBG interrogator (>80 nm) but it is very important in order to analyze the short circuit response time properly. According to Zhang et al. [4] the fundamental frequency of the axial force during the surge event is twice the frequency of fundamental current.

**Figure 10:**
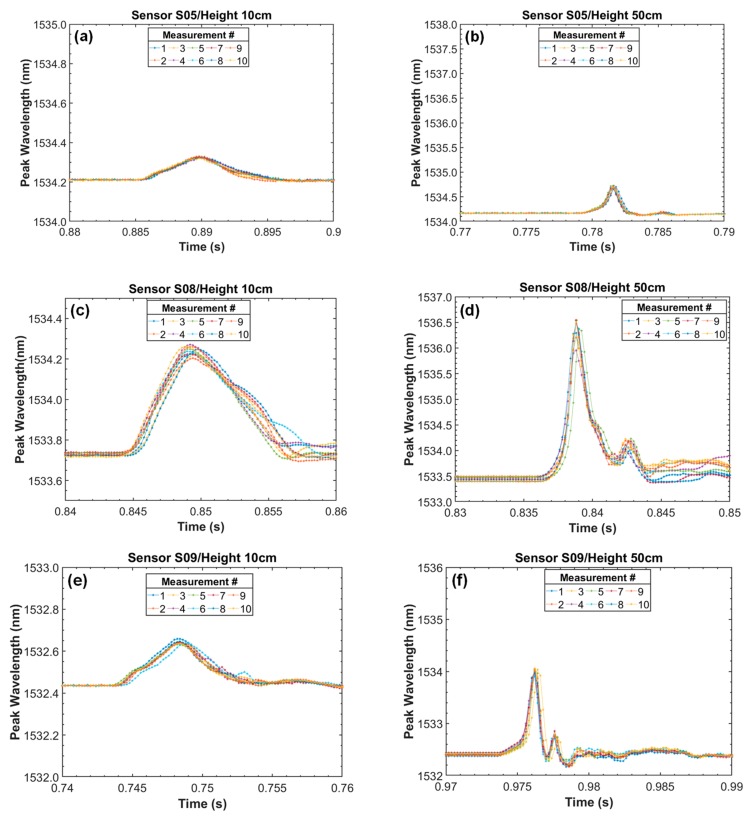
Results of dynamic tests performed using free fall height, for (**a**) TB (S05) 10 cm, (**b**) TB (S05) 50 cm, (**c**) PEEK (S08) 10 cm, (**d**) PEEK (S08) 50 cm, (**e**) PEEK (S09) 10 cm and (**f**) PEEK (S09) 50 cm.

Figure 10 shows the results of dynamic measurements of the three sensors when dropping the weight from a height of 10 cm and 50 cm. It is noted in Figure 10 that the wavelength peak amplitude increased and the time response period decreased with the impact height.

It is also possible to observe in Figure 10 that the TB material sensor was less sensitive compared to those made of PEEK, as similarly observed in the previous static tests. To obtain a sensor sensitivity curve, the average peak wavelength amplitude obtained from 10 measurements was calculated.

To obtain a peak wavelength curve as a function of the impact force, Equation (3) was used. The impact time was measured for each height and an average of the obtained values was calculated. Knowing the impact time value (temporal width at –20 dB from the peak), the force was calculated and its values are presented in Table 3.

**Figure 11:**
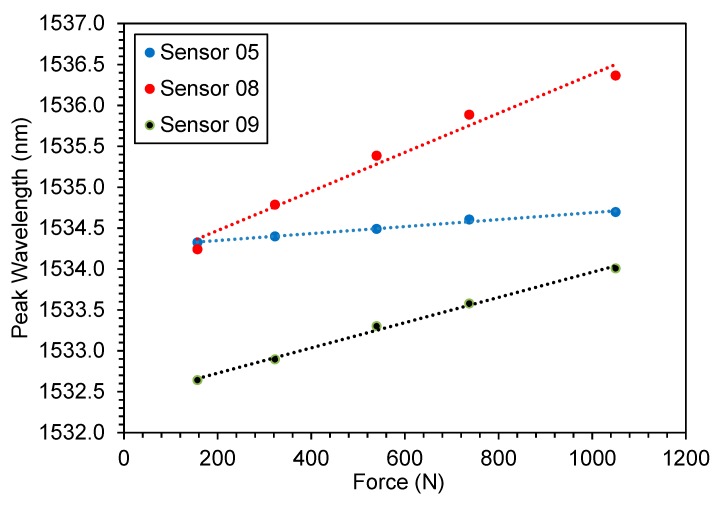
FBG peak wavelength curve as a function of the free fall height of the weight thrown against sensors S05, S08 and S09.

A peak wavelength curve as a function of impact force is shown in Figure 11. The sensitivities of the three sensors were 0.426 pm/N for S05 (TB), 2.39 pm/N for S08 and 1.54 pm/N for S09 (PEEK sensors). Thus, the sensitivity of the best PEEK sensor was 5.61 times higher than the TB sensor, same order of magnitude obtained in the static results.

Finally, it is possible to estimate the frequency bandwidth of the sensing system (sensor plus interrogator) using the expression of Equation (4) [27]:

(4) $$B=\frac{0.35}{t_{s}},$$

where t_s_ is the rising time of the impulse response of the sensor measured between 10% to 90% level of the steady maximum value. The frequency bandwidth, and thus the rising time, of the sensing system was limited by its material properties such as Young modulus and Poisson ratio as well as the by the interrogator sweep frequency (5 kHz in this case). Considering the rising time value of sensor S08 for 50 cm height (1.2 ms) the calculated bandwidth was 291.6 Hz. Since the fundamental frequency of the axial force in current surge event is 120 Hz the estimated sensing system bandwidth of 291.6 Hz is enough to measure the transitory events in the transformer.

## 4. Discussion

In this work it was evaluated the performance of FBG pressure sensors developed for monitoring the static and dynamic pressures in high voltage winding transformers during events such as short-circuit and inrush current. Two types of sensors packaging materials were evaluated in laboratory: PEEK and TB. 

It is important to note that FBG, PEEK and TB are insulating materials proper to operate in high voltage environments, it is necessary to ensure its chemical compatibility with the used mineral oil. TB and FBG have been already used in transformers as insulating material and as commercially available temperature sensors. The PEEK material was tested in an independent chemical laboratory to confirm its applicability. The results indicated complete compatibility of the PEEK material with the mineral oil, following the Brazilian standard [28].

The sensors were stressed in typical short circuit winding pressures. In addition, the time response of the sensors was evaluated along with the 5 kHz interrogation system for the analysis of mechanical effects similar to those produced in short circuit events. The result pointed that FBG pressure sensors using PEEK and TB materials were excellent choices for transformer winding monitoring, demonstrating linear responses for applied pressures in two evaluated ranges from 800 N to 1500 N and from 3000 N to 10,000 N with good repeatability. PEEK sensors exhibited better sensitivities when compared with TB sensor; however, the last sensor presented better repeatability, especially in the higher stress regime. The dynamical responses of the sensors showed excellent repeatability for both sensors with smaller sensitivity of TB in comparison with PEEK, which was up to 5.61 times more sensitive. In addition, the bandwidth of the sensing system (around 290 Hz) was enough to measure the transitory events in the transformer.

The future directions of this research will encompass transformer instrumentation with these sensors in order to assess their performance in a real application. In this real transformer installation, it is necessary to install a temperature compensation FBG sensor in order to avoid typical temperature and strain cross sensitivity issues.

## Figures and Tables

**Table 1:** Some typical polyether ether ketone (PEEK) [18], transformerboard (TB) [19] and optical fiber physical properties [23,24,25].

Properties	Unit	TB	PEEK	Fiber
Density	Kg/m³	1250	1320	2200 [23]
Tensile strength	MPa	97	92	3500 [23]
Maximum Temperature	°C	105	250	300 * [24]
Dielectric strength	kV/mm	14.5 (in oil)	20	47–67 [25]

* Polyimide coating.

**Table 2:** Sensor dimension values for PEEK and TB.

Dimensions (mm)	TB	PEEK
D_disk_	31	31
h_disk_	8.8	8.8
h_block_	19	17.4
h_elipse_	11	7
h_1_	4.2	6.4
D_elipse_	22	22
w_block_	50	50
D_block_	50	50

**Table 3:** Conversion between height and impact force.

Height (cm)	Impact Time (ms)	Calculated Force (N)
10	10.8	155.6
20	7.7	309.9
30	5.4	538.9
40	4.6	730.4
50	3.7	1024.5

## Supplementary Materials

The following video is available online at https://www.mdpi.com/1424-8220/19/22/4877/s1. V1: The procedure of static tests.
